# Adverse Associations of both Prenatal and Postnatal Exposure to Organophosphorous Pesticides with Infant Neurodevelopment in an Agricultural Area of Jiangsu Province, China

**DOI:** 10.1289/EHP196

**Published:** 2016-05-06

**Authors:** Ping Liu, Chunhua Wu, Xiuli Chang, Xiaojuan Qi, Minglan Zheng, Zhijun Zhou

**Affiliations:** 1 School of Public Health/MOE Key Laboratory of Public Health Safety/WHO Collaborating Centre for Occupational Health (Shanghai), Fudan University, Shanghai, China; 2 Zhejiang Provincial Center for Disease Control and Prevention, Hangzhou, China; 3 National Shanghai Center for New Drug Safety Evaluation & Research (NCDSER), Shanghai, China

## Abstract

**Background:**

Prenatal exposure to organophosphorous (OP) pesticides has been found to be associated with adverse effects on child neurodevelopment, but evidence on potential effects induced by both prenatal and postnatal OP exposure in infants is limited.

**Objectives:**

Our aim was to investigate the associations of both prenatal and postnatal OP exposure with birth outcomes and infant neurodevelopment.

**Methods:**

Exposure to OP in 310 mother–infant pairs was assessed by measuring dimethylphosphate (DM), diethylphosphate (DE), and total dialkylphosphate (DAP) metabolites in urines from pregnant women and their children at 2 years of age. The Gesell Developmental Schedules was administered to examine neurodevelopment of 2-year-old children.

**Results:**

Based on the Gesell Developmental Schedules, the proportions of children with developmental delays were < 6%. Adverse associations between head circumference at birth and prenatal OP exposure were demonstrated. Both prenatal and postnatal OP exposure was significantly associated with increased risk of being developmentally delayed. Specifically, odds ratio (OR) value for prenatal DEs was 9.75 (95% CI: 1.28, 73.98, p = 0.028) in the adaptive area, whereas in the social area, OR values for postnatal DEs and DAPs were 9.56 (95% CI: 1.59, 57.57, p = 0.014) and 12.00 (95% CI: 1.23, 117.37, p = 0.033), respectively. Adverse associations were observed only in boys, not in girls.

**Conclusions:**

Both prenatal and postnatal OP exposure may adversely affect the neurodevelopment of infants living in the agricultural area. The present study adds to the accumulating evidence on associations of prenatal and postnatal OP exposure with infant neurodevelopment.

**Citation:**

Liu P, Wu C, Chang X, Qi X, Zheng M, Zhou Z. 2016. Adverse associations of both prenatal and postnatal exposure to organophosphorous pesticides with infant neurodevelopment in an agricultural area of Jiangsu Province, China. Environ Health Perspect 124:1637–1643; http://dx.doi.org/10.1289/EHP196

## Introduction

Organophosphorous (OP) pesticides—toxicants known to disrupt neurologic development—are extensively used in China and worldwide (González-Alzaga et al. 2014). Developmental OP exposure could inhibit DNA synthesis, reduce the numbers of neural cells, and lead to abnormalities of synaptic activity within a critical window of neurodifferentiation (Crumpton et al. 2000; Slotkin and Seidler 2012). Developing fetuses and infants are thought to be highly susceptible to OP exposure, due to the ready maternal–fetal transfer of OP pesticides through the placenta, and the immaturity in neurologic development and metabolic detoxification pathways (Kousba et al. 2007). In daily life, young children may experience long-term, low doses of OP pesticides through historical home use, child care environment, and residues in food (Morgan et al. 2005). Thus, children have the potential risk of adverse neurodevelopmental effects induced by OP exposure, even at doses that do not exceed the degree of cholinesterase inhibition necessary to produce systemic toxicity (Crumpton et al. 2000).

Exposure to OP pesticides has been found to be negatively associated with child growth and neurodevelopment outcomes. Several birth cohort studies were performed to investigate the neurodevelopmental toxicity of prenatal and/or postnatal exposure to OP pesticides among children, which were reviewed by González-Alzaga et al. (2014). Prenatal exposure to OP pesticides was associated with decreased fetal growth (Perera et al. 2003), shortened gestation (Eskenazi et al. 2004), poorer neurobehavioral development among infants and toddlers (Engel et al. 2007; Eskenazi et al. 2007; Rauh et al. 2006) and in preschoolers (Bouchard et al. 2011). However, the associations between postnatal exposure to OP pesticides and child neurodevelopment were not definitive. Adverse associations were reported between postnatal OP exposure and behavioral problems, cognitive deficits, longer reaction time, and other neurodevelopmental disorders in children across cross-sectional studies (Bouchard et al. 2010; Rohlman et al. 2005), whereas no associations were found between postnatal OP exposure and children’s motor or cognitive function in several studies (Bouchard et al. 2011; Guodong et al. 2012). What was more complex was that the elevated postnatal OP exposure was associated with increases of cognitive scores (Eskenazi et al. 2007). Overall, few studies discussed health effects of both prenatal and postnatal exposure to OP pesticides on child neurodevelopment.

In our previous studies, pregnant women and their infants had shown widespread pyrethroid insecticides exposure in Sheyang County, Jiangsu Province (Qi et al. 2012; Wu et al. 2013). In the present study, 310 2-year-old children of the registered women in the previous study were enrolled to assess the associations of both prenatal and postnatal urinary OP metabolite levels with children’s birth outcomes and neurodevelopment at 2 years of age.

## Materials and Methods

### Study Subjects

A total of 310 mother–infant pairs who lived in Sheyang County were enrolled into the present study. Sheyang County, located north of Jiangsu Province, China, is known as a high-quality cotton and rice production region. Agricultural land area is estimated to be about 129,333 ha, with approximately 2,100 tons of pesticides applied annually to control insect pests and plant diseases (Liu et al. 2013).

During June 2011–January 2012, 405 infants whose mothers were from our previous study (Qi et al. 2012) initially participated in the present investigation and visited Sheyang Maternal and Child Health Care Centre. Subjects who volunteered to participate in the study signed an informed consent form and agreed to donate urine samples. This study was carried out with the permission of the Health Bureau of Sheyang County and the Ethics Committees of Fudan University. We excluded 54 children with missing prenatal urine samples, 33 children without adequate postnatal urine volume of 15 mL, and 8 children who did not complete the questionnaires. Mother–infant pairs included in this analysis (*n* = 310) did not differ significantly (*p* > 0.05) from the initial recruited subjects (*n* = 405) on all attributes of interest, including maternal age, maternal education, maternal work status, smoking status, family socioeconomic information, and children’s neurodevelopmental scores.

### Questionnaire Data

Mothers were interviewed within the first week of delivery by trained interviewers. Information on maternal health, maternal education, household income, occupational history, maternal smoking, alcohol consumption, and history of residential pesticide use was collected and described in a previously published study (Qi et al. 2012).

Questionnaires for children at 2 years of age were administered to the accompanying adults (parents or grandparents) at the Health Care Centre. The questionnaire elicited demographic and socioeconomic information including child behavior habits, feeding pattern, household characteristics (e.g., location of dwelling and house quality), present parental occupation, and residential pesticide use (Liu et al. 2014).

### Urine Sample Collection

Maternal urine samples were collected prior to delivery (Qi et al. 2012), and children’s urine samples were collected at the Health Care Centre (Liu et al. 2014). The urine samples were then transferred to the high-density polypropylene centrifuge tubes (Corning Inc.). All samples were immediately stored at –20°C, shipped in a frozen state to the laboratory, and kept frozen at –80°C until analysis. These urine samples were measured for six nonspecific dialkylphosphate (DAP) metabolites and creatinine.

### OP Exposure Assessment

Six DAP metabolites were measured by gas chromatography–mass spectrometry based on our previous method (Wu et al. 2006) with slight modification. Those measured were three dimethyl (DM) phosphate metabolites—dimethylphosphate (DMP), dimethylthiophosphate (DMTP), dimethyldithiophosphate (DMDTP); and three diethyl (DE) phosphate metabolites—diethylphosphate (DEP), diethylthiophosphate (DETP), and diethyldithiophosphate (DEDTP). The limits of detection (LODs) in our study were 0.5 μg/L for DMP, DMTP, DMDTP, and DEP, and 0.25 μg/L for DETP and DEDTP, defined as a signal-to-noise ratio of 3. The mean relative recoveries for six DAP metabolites ranged from 90.7% to 116.9%, with coefficients of variation (CV) ranging from 5.7% to 10.9%.

Creatinine concentrations were measured using ELx800 Universal Microplate Reader (wavelength 340–750 nm; BIO-TEK). Urinary DAP concentrations were presented both in μg/L and in μg/g Cre, adjusted based on creatinine levels.

### Measures of Fetal Growth

Information on newborns was obtained from hospital medical records, including gestational age at birth, infant sex, head circumference, and birth weight and length. Infant ponderal index (PI), a measure of proportionality of growth, was calculated as (birth weight in kilograms)/(length in meters)^3^. PI malnutrition of Chinese infant was defined as PI < 20.5 (Ying 2008). Low birth weight was defined as < 2,500 g, and fetal macrosomia was defined as > 4,000 g. Preterm delivery was defined as birth at < 37 completed weeks of gestation.

### Child Neurodevelopment Assessment

The Gesell Developmental Schedules (GDS) was designed to provide a neurologic and intellectual evaluation of the child at the time of testing (Gesell and Amatruda 1941). The Chinese version of the GDS for 0- to 3-year-old children was revised by the Beijing Mental Development Cooperative Group (1985). It was used in China to assess infant intellectual development for abnormality after exposure to polycyclic aromatic hydrocarbons (Tang et al. 2014), and also clinically used to diagnose developmental delay of infants with brain damage syndrome (Liu et al. 2016), hyperthyroidism (Huo et al. 2011), and tuberous sclerosis-related west syndrome (Liu et al. 2012).

The GDS items are grouped into four neurobehavioral domains, namely motor, adaptive, language, and social areas. Specifically, motor behavior includes balance, walking, and hand control (5 items); adaptive behavior includes imitation, discriminative performance, and perception (7 items); language behavior was assessed by means of vocabulary, word comprehension, and conversation (6 items); and social behavior includes reactions to persons, personal habits, and acquired information (10 items).

According to test items, one pediatrician estimated a maturity age of neurodevelopment (expressed in months) for each of the four specific domains. The maturity age can be used to generate a developmental quotient (DQ), which is the maturity age divided by the chronologic age, multiplied by 100. Then children can be classified as normal (DQ scores > 84), moderate delay (DQ scores, 70–84), or severe delay (DQ scores < 70) based on predetermined cut points (Gesell et al. 1974). In the present study, the GDS was conducted by four trained pediatricians who completed formal training with qualifications at Xin Hua Hospital Affiliated to Shanghai Jiao Tong University School of Medicine. Each subject was examined by one pediatrician for the four domains. To minimize both interexaminer and intraexaminer variability, every effort was made to maximize reliability in scoring by performing standardized training procedures and regular self-checking.

### Data Analysis

The questionnaire data were input into Epidata3.1 software (EpiData Association, Odense, Denmark; http://www.epidata.dk). Multiple linear regressions were conducted with SPSS version 19.0 (IBM Corp.). Logistic regression analyses and seemingly unrelated regression (SUR) estimation were performed with STATA version 12.0 (StataCorp). Generalized additive models (GAMs) were performed with SAS version 9.3 (SAS Institute Inc.). An imputed value of LOD divided by the square root of 2 was assigned to levels below the detection limit (Hornung and Reed 1990). The 0.05 level of probability was used as the criterion of significance. All analyses were conducted on non–creatinine-adjusted values. Models were re-run with creatinine-adjusted values in sensitivity analyses. The separate multiple regression and logistic regression models were used for DM, DE, and total DAP concentrations, respectively.

For prenatal exposure, we examined the associations of maternal DAP concentrations with birth outcomes (head circumference, birth weight and length, neonatal PI) and DQ scores at 2 years of age after adjustment for a range of confounding factors as the covariates. Covariates were included in the prenatal multiple regression models if they related to neurodevelopment in the literature, associated with any outcomes (*p* < 0.10) or changed the coefficients of urinary DAP concentrations by ≥ 10% (Marks et al. 2010), along with known potential confounders from other reports. The prenatal models included continuous covariates for multiple regression models as follows: maternal age, gestational age, pregnancy weight gain, and maternal body mass index (BMI) before pregnancy; categorical covariates such as parity, delivery mode, child’s sex, passive smoking (yes, no), maternal work status (agricultural and factory work, other work), paternal work status (agricultural and factory work, other work), family annual income (< US$5,000, ≥ US$5,000), maternal education (< high school, ≥ high school), cord blood lead values (< median, > median), sampling season (before September, after September), and inhabitation (town, suburb, and countryside).

For postnatal exposure, we examined the cross-sectional association of DQ scores with DAP concentrations measured in children’s urine collected at 2 years of age. We retained the following variables as covariates for postnatal multiple regression model: child’s sex, feeding pattern (artificial feeding, other pattern), inhabitation, child hand-to-mouth behaviors (frequent, few), whether families lived near (100 m distance) plantations or green parks, whether chipped paint fell from the wall in homes, passive smoking, whether family used indoor insecticides within 1 year, whether family used mosquitocides within 1 year, at least one farmer in household, family annual income, maternal work status, paternal work status, maternal education, and sampling season.

We used logistic regression to analyze the associations between maternal DAP concentrations and the probability of being GDS developmentally delayed with the same covariates as multiple regression models. A score of 84 is the cutoff point for determining normal or being developmentally delayed in the GDS. The associations between children’s DAP concentrations and probability of being developmentally delayed were also assessed.

To explore possible joint effects between pre- and postnatal OP exposure, we built the combined multiple regression models and included an interaction term for prenatal DAP concentrations × postnatal DAP concentrations. No evident joint effect of prenatal and postnatal OP exposure was observed in the present study (*p* > 0.15), and thus the combined models are not presented.

SUR was used to compare effect estimates of urinary DAPs measured in the prenatal versus postnatal periods (Bouchard et al. 2011). We ran GAMs by fitting splines to evaluate the shape of the dose–effect relationship, test the linearity assumption, and investigate potential thresholds while controlling for covariates. Because no nonlinear dose–effect relationships were found (*p* > 0.10), linear models were still used.

Finally, in sensitivity analyses, we considered whether controlling for growth parameters (i.e., head circumference, birth weight, and birth length) and other suspected pollutants (i.e., pyrethroid pesticides, lead, and cadmium) altered our present results. The interaction between pyrethroid insecticides and OP pesticides was also examined, using *p* < 0.15 for the interaction term (Marks et al. 2010). We re-ran models using log-transformed creatinine-adjusted DAP metabolites. Furthermore, because the effect of OPs was found to vary by sex (González-Alzaga et al. 2014), we tested for interactions between DAP concentrations and child’s sex to determine whether associations of DAP concentrations with DQ scores differed between boys and girls. All models were explored in all study subjects and also stratified by sex.

## Results

The sociodemographic characteristics of mother–infant pairs are listed in Table 1. The mean (± SD) duration of gestation was 39.3 ± 1.0 weeks; mean birth weight was 3.52 ± 0.44 kg, with 1.3% of low birth weight and 14.2% of fetal macrosomia; mean body length was 51.2 ± 2.4 cm; mean head circumference was 34.6 ± 1.5 cm; and mean PI was 26.33 ± 3.65 kg/m^3^, with 4.5% of PI malnutrition.

**Table 1:** General information of mother–infant pairs (*n* = 310) in Sheyang County, China.

General information	*n* (%)
Sex of infant
Male	178 (57.4)
Female	132 (42.6)
Maternal age (years)
< 25	162 (52.3)
25–35	123 (39.7)
> 35	25 (8.1)
Maternal education level
≤ Elementary school	26 (8.4)
Junior middle school	201 (64.8)
≥ High school	83 (26.8)
Maternal work status during pregnancy
Agricultural work	11 (3.6)
Factory work	117 (37.7)
Other work	182 (58.7)
Feeding patterns^*a*^
Breastfeeding	14 (4.5)
Mixed feeding	25 (8.1)
Artificial feeding	271 (87.4)
Child hand-to-mouth activities^*b*^
Frequent	152 (49.0)
Few	158 (51.0)
Residence
Town	71 (22.9)
Suburb	97 (31.3)
Countryside	142 (45.8)
Homes near plantations or green parks (100 m distance)
Yes	203 (65.5)
No	107 (34.5)
Indoor insecticides use within 1 year
Yes	74 (23.9)
No	236 (76.1)
Lived in family with smokers
Yes	159 (51.3)
No	151 (48.7)
At least one farmer in household
Yes	130 (41.9)
No	180 (58.1)
Family annual income during pregnancy
≤ US$5,000	144 (46.5)
> US$5,000	166 (53.5)
Chipped paint falling from the wall in homes
Yes	23 (7.4)
No	287 (92.6)
^***a***^Mixed feeding: combining breast feeding and other food or formula milk; artificial feeding: feeding of a baby with food (formula milk, fruits or normal diet) other than breast feeding. ^***b***^Frequent: number of hand-mouth contacts/hour > 10 reported by caregivers; few: number of hand-mouth contacts/hour ≤ 10 reported by caregivers.	

The distributions of maternal and child urinary DAP concentrations are listed in Table S1. The developmental scores and proportions delayed based on the GDS are also presented (see Table S2). The mean DQ scores ± SD in the motor, adaptive, language, and social areas were 99.28 ± 8.83, 96.17 ± 8.21, 95.69 ± 9.22, and 96.97 ± 8.31, respectively. The proportions of developmental delay in four domains were 1.61, 3.23, 5.81, and 3.55%, respectively.

Adverse associations between head circumference at birth and measures of prenatal OP exposure were demonstrated (see Table S3). Specifically, for every 10-fold increase (i.e., one log-unit increase) in prenatal DAP concentrations, a 0.67-cm (*p* = 0.017) decrease of head circumference in all subjects was found. Among boys, for every 10-fold increase in prenatal DE concentrations, a 0.65-cm (*p* = 0.043) decrease of head circumference was observed; for every 10-fold increase in prenatal DAP concentrations, a 1.04-cm (*p* = 0.015) decrease of head circumference was found.

Prenatal DE concentrations were significantly associated with increased risk of being developmentally delayed in the adaptive area (Table 2). Odds ratio (OR) value for prenatal DEs was 9.75 [95% confidence interval (CI): 1.28, 73.98, *p* = 0.028]. Among boys, prenatal DE concentrations were also associated with increased risk of being developmentally delayed in the adaptive area (OR = 26.41; 95% CI: 1.25, 557.40, *p* = 0.035). Additionally, we found evidence of effect modification by sex in the language and social area (*p* < 0.15).

**Table 2:** Results of logistic regression analysis of GDS developmental delay and maternal DAP concentrations during pregnancy (*n* = 310).*^a^*

Domains	OR^*b*^ (95% CI)	*p-*Value	*p*-Value for interaction^*c*^	Male		Female	
				OR (95% CI)	*p-*Value	OR (95% CI)	*p-*Value
Motor area
DMs	0.95 (0.07, 13.10)	0.969	0.691	6.60 (0.12, 361.11)	0.355	0.16 (0.01, 4.88)	0.297
DEs	0.63 (0.06, 6.91)	0.703	0.918	1.06 (0.00, 240.38)	0.984	0.50 (0.02, 13.55)	0.680
DAPs	0.64 (0.03, 14.97)	0.784	0.617	56.05 (0.02, 160127.90)	0.321	0.17 (0.00, 14.79)	0.439
Adaptive area
DMs	1.22 (0.19, 7.88)	0.833	0.470	2.31 (0.11, 50.67)	0.596	0.83 (0.07, 9.90)	0.885
DEs	9.75 (1.28, 73.98)	0.028	0.486	26.41 (1.25, 557.40)	0.035	3.98 (0.20, 77.95)	0.363
DAPs	9.69 (0.81, 115.48)	0.072	0.303	48.32 (0.89, 2611.38)	0.057	3.09 (0.11, 86.08)	0.506
Language area
DMs	0.92 (0.22, 3.87)	0.911	0.692	0.85 (0.10, 7.36)	0.884	1.13 (0.13, 9.69)	0.911
DEs	1.93 (0.40, 9.29)	0.414	0.047	7.77 (0.73, 82.22)	0.089	0.83 (0.09, 7.99)	0.873
DAPs	1.75 (0.25, 12.51)	0.575	0.080	6.14 (0.34, 112.15)	0.221	0.87 (0.05, 13.85)	0.923
Social area
DMs	0.81 (0.12, 5.65)	0.833	0.749	0.47 (0.02, 11.28)	0.644	0.16 (0.00, 41.47)	0.518
DEs	1.99 (0.31, 12.69)	0.466	0.137	3.42 (0.27, 43.34)	0.342	1.67 (0.01, 347.50)	0.850
DAPs	1.70 (0.14, 20.06)	0.675	0.271	2.33 (0.07, 80.06)	0.640	1.52 (0.00, 3547.79)	0.916
Average scores
DMs	0.31 (0.04, 2.29)	0.249	0.190	0.42 (0.02, 10.46)	0.596	0.04 (0.00, 2.33)	0.122
DEs	2.01 (0.20, 20.30)	0.555	0.677	2.14 (0.06, 73.80)	0.674	7.25 (0.14, 38526.89)	0.651
DAPs	1.00 (0.07, 14.73)	0.998	0.534	0.21 (0.00, 34.59)	0.553	0.18 (0.00, 6426.46)	0.745
^***a***^Adjusted for maternal age, gestational age, pregnancy weight gain, maternal BMI before pregnancy, parity, delivery mode, child’s sex, passive smoking, maternal work status during pregnancy, paternal work status, family annual income, maternal education, cord blood lead values, sampling season, and residence. ^***b***^OR values presented assume that there is no interaction by child’s sex. ^***c***^Interaction between DAP concentrations and child’s sex.							

Postnatal DE and DAP concentrations were significantly associated with increased odds of being developmentally delayed in the motor and social area, especially among boys (Table 3). In the motor area, the OR for postnatal DEs was 13.20 (95% CI: 1.35, 128.80, *p* = 0.026), whereas postnatal DEs (*p* = 0.014) and DAPs (*p* = 0.036) were both associated with increased risk of being developmentally delayed among boys. In the social area, ORs for postnatal DEs and DAPs were 9.56 (95% CI: 1.59, 57.57, *p* = 0.014) and 12.00 (95% CI: 1.23, 117.37, *p* = 0.033), respectively. Similarly, the boys with elevated DE concentrations had increased odds of being developmentally delayed (*p* = 0.029). We also found evidence of effect modification by sex in the motor and adaptive area (*p* < 0.15).

**Table 3:** Results of logistic regression analysis of GDS developmental delay and child DAP concentrations (*n* = 310).*^a^*

Domains	OR^*b*^ (95% CI)	*p-*Value	*p*-Value for interaction^*c*^	Male		Female	
				OR (95% CI)	*p-*Value	OR (95% CI)	*p-*Value
Motor area
DMs	4.28 (0.46, 40.25)	0.203	0.769	5.75 (0.27, 121.00)	0.260	2.94 (0.11, 78.73)	0.520
DEs	13.20 (1.35, 128.80)	0.026	0.067	263.76 (3.07, 22633.10)	0.014	1.70 (0.08, 35.70)	0.732
DAPs	14.60 (0.87, 246.22)	0.063	0.162	145.13 (1.37, 15336.35)	0.036	2.27 (0.07, 74.91)	0.646
Adaptive area
DMs	2.64 (0.52, 13.40)	0.240	0.748	2.99 (0.24, 36.58)	0.392	3.04 (0.31, 29.76)	0.339
DEs	3.28 (0.67, 16.08)	0.143	0.131	17.21 (0.74, 402.00)	0.077	1.35 (0.19, 9.38)	0.761
DAPs	4.43 (0.68, 28.86)	0.120	0.275	16.65 (0.48, 574.72)	0.120	2.28 (0.23, 22.72)	0.483
Language area
DMs	2.30 (0.63, 8.34)	0.206	0.223	0.87 (0.10, 7.20)	0.894	4.13 (0.52, 32.56)	0.178
DEs	1.74 (0.54, 5.55)	0.351	0.209	0.84 (0.16, 4.36)	0.835	3.61 (0.50, 26.04)	0.203
DAPs	2.10 (0.50, 8.79)	0.309	0.209	0.65 (0.07, 5.81)	0.698	4.35 (0.46, 41.31)	0.201
Social area
DMs	3.96 (0.72, 21.78)	0.114	0.931	4.17 (0.38, 45.42)	0.242	3.71 (0.10, 132.56)	0.472
DEs	9.56 (1.59, 57.57)	0.014	0.560	57.36 (1.51, 2182.03)	0.029	10.27 (0.21, 502.61)	0.240
DAPs	12.00 (1.23, 117.37)	0.033	0.649	53.13 (0.87, 3244.01)	0.058	10.10 (0.10, 1056.58)	0.330
Average scores
DMs	2.61 (0.48, 14.04)	0.265	0.615	3.65 (0.28, 46.85)	0.320	1.53 (0.12, 20.12)	0.744
DEs	2.95 (0.63, 13.91)	0.171	0.179	12.86 (0.74, 223.33)	0.079	0.76 (0.06, 9.09)	0.830
DAPs	3.36 (0.50, 22.53)	0.211	0.238	2.84 (0.89, 6.57)	0.136	1.04 (0.07, 16.41)	0.981
^***a***^Adjusted for child’s sex, feeding pattern, residence, child hand-to-mouth contacts, whether families lived nearby plantations or green parks, whether chipped paint falling from the wall in homes, passive smoking, whether family used indoor insecticides within 1 year, whether family used mosquitocides within 1 year, at least one farmer in household, family annual income, maternal work status, paternal work status, maternal education, and sampling season. ^***b***^OR values presented assume that there is no interaction by child’s sex. ^***c***^Interaction between DAP concentrations and child’s sex.							

No statistically significant associations between DAP concentrations and DQ scores were observed in multiple regression models (see Table S4 and Table S5). The comparison of the associations between DQ scores and total DAP concentrations measured in the prenatal versus postnatal periods is shown in Table S6. The effect coefficients of OP exposure measured in maternal urines were not significantly different from those for children’s levels (*p* > 0.05).

For the sensitivity analyses, adjusting OP metabolites by creatinine, growth parameters, and additional prenatal chemical exposures did not substantially change our primary results. No suspected pollutants were found to confound or interact with the DAP concentrations. In addition, adjusting DAP metabolites by creatinine only made some coefficients become slightly stronger and some weaker.

## Discussion

Our findings suggested that prenatal exposure to OP pesticides was adversely associated with head circumference at birth and with neurodevelopment in children at 2 years of age. Furthermore, we also found that postnatal DAP concentrations in 2-year-old children were associated with being developmentally delayed in the motor and social area. This study added to the weight of evidence that there were significant associations between OP exposure and developmental deficits in infants.

In the present study, head circumference at birth was negatively associated with measures of prenatal DE and DAP concentrations. This finding was consistent with Wolff et al.’s (2007) study, which suggested that prenatal DAP metabolites were weakly associated with a small decrease in head circumference (0.26 cm; *p* = 0.045). Similarly, Berkowitz et al. (2004) found that *in utero* chlorpyrifos exposure was associated with a significant but small reduction in head circumference among mothers who exhibited low paraoxonase (PON1) activity. In contrast, Perera et al. (2003) only found decreased birth weight and length in association with maternal OP exposure in pregnant women. Eskenazi et al. (2004) observed increased but not decreased head circumference in relation to prenatal DAP concentrations. Our median levels of prenatal DM, DE, and DAP concentrations—128.16, 134.88, and 295.80 nmol/L, respectively—were higher than those reported by Eskenazi et al. (101, 22, and 136 nmol/L, respectively). The higher prenatal exposure to OP pesticides might contribute to a more evident detrimental effect on fetal growth. In addition, because head circumference of newborns has been shown to correlate with brain size (Cooke et al. 1977) and associate with cognitive function in later life (Broekman et al. 2009), head circumference at birth may be a confounder or potential factor on the causal pathway between prenatal OP exposure and developmental outcomes at 2 years of age. Nevertheless, adverse effects of prenatal OP exposure still persisted after adjusting for head circumference in our study.

Adverse associations between prenatal exposure to OP pesticides and children’s neurodevelopment were reported in several studies (e.g., Bouchard et al. 2011; Engel et al. 2007). Similarly, we found prenatal DE concentrations were associated with increased risk of being developmentally delayed in the adaptive area. On the other hand, children’s OP metabolites were also found to be adversely associated with cognitive abilities (Rohlman et al. 2005). Likewise, we observed that DAP concentrations measured in children were associated with being developmentally delayed in the motor and social area. However, several investigations reported different results related to associations between OP exposure and children’s neurodevelopment. For instance, Eskenazi et al. (2007) found the elevated postnatal DAP concentrations were associated with an increase in the Mental Development Index (MDI) based on the Bayley Scales of Infant Development (BSID). Guodong et al. (2012) used the same GDS as we used in our study but observed no associations between child urinary OP metabolite levels and DQ scores among young children of Shanghai city. The geometric mean values of DE and DAP metabolites in our study were higher than those of Guodong et al. (2012), which indicates that OP exposure levels in the agricultural environment may be higher than those in an urban community. In sum, the disparities of results may be attributable to differences in exposure measurements, neurodevelopmental assessment methods, and study population of different investigations.

In the present investigation, children with developmental deficits had immature or compromised performances compared with normal children. The 2-year deficits in the present report could provide a moderate predictor for subsequent intelligence. According to the study of Zhou et al. (2004), there was a significant correlation between developmental assessment at 6–12 months on the GDS and mental development at 6–7 years on the Chinese version of the Wechsler Intelligence Scale for Children (WISC) (*p* < 0.01). Moreover, the developmental deficits emerged by 2 years of age might also be relating to school performances. As Sullivan and Margaret (2003) observed, children with 4-year motor delay had lower academic achievement scores and higher rates of school service use at age 8 years, including additional lessons, physical therapy, occupational therapy, and the like. On the other hand, the compromised performances may also correlate with early education and surroundings during infancy. It was noteworthy that DQ scores of infants who accepted early education before 3 years old were significantly higher than nonaccepted ones (Li et al. 2008). Although our primary results were not substantially changed after adjusting for maternal education, the potential effects of early education were still non-negligible.

It is generally known that neurodevelopmental effects caused by exposure to a neurotoxic compound during a critical window of development might manifest later in time, because cascading development processes are continuously taking place (Rice and Barone 2000). This kind of time delay had been observed in several epidemiological studies. In a study of 2-year-old children, no association was found between prenatal DAPs and attention or attention deficit/hyperactivity disorder (ADHD)–related problems (Eskenazi et al. 2007). However, in the same cohort prenatal DAPs were associated with increased odds of attention problems and poorer attention scores at 3.5 and 5 years of age (Marks et al. 2010). Moreover, these associations appeared to be somewhat stronger at 5 years than at 3.5 years. The subjects in the present study were probably too young at 2 years of age to manifest more neurodevelopmental problems. Therefore, impacts of OP exposure on subsequence developmental problems deserve further investigation.

In the present study, only boys showed adverse associations of both prenatal and postnatal OP exposure with being developmentally delayed, which suggested that OP effects differed by sex. Our results were similar to Horton et al.’s (2012) study, which reported that males experienced a greater decrement in working memory than females following prenatal OP exposure. Several biological factors may contribute to such differential vulnerability to OP pesticides by sex, including differences in OP metabolism, repair processes of damage, and hormonal differences. The first potential explanation was that males had a higher rate of hepatic activation of OP and produced more corresponding oxygen analogs than females (Sultatos 1991). Second, the inhibition of cholinesterase was greater in males than in females after postnatal exposure (Dam et al. 2000). Further, several commonly used OP pesticides are endocrine disruptors. For example, chlorpyrifos could inhibit androgen biosynthesis (Viswanath et al. 2010), sex-differentially affect levels of thyroid-stimulating hormones (De Angelis et al. 2009), and alter the activity of neurohormones implicated in the modulation of social and affective responses, with males presenting more intense effect (Tait et al. 2009). Additionally, males have a slower rate of cortical development than females, making the male brain susceptible to insult for a longer period (Taylor 1969). Besides the biological factors, the social factors may also play an important role in sex differences. As Beamer et al. (2008) reported, boys had higher contact frequencies with the ground and toys, whereas girls had longer contact duration with clothes and other objects. Thus, the differences in activities between boys and girls could lead to differences in their exposure to OP pesticides.

One highlight was that we observed adverse neurodevelopment effects on infants in relation to both prenatal and postnatal OP exposure, after adjustment for important modifying factors including maternal education, family socioeconomics, and exposure to other known neurotoxicants. The sensitivity analysis showed that the effects of OP pesticides were not attributable to the effects of the known confounders. However, so many statistical tests repeatedly performed might increase the probability of false positives. To control the type 1 error, the significance level for each test must be more stringent, but this technique may raise the rate of false negatives, failing to identify the potential detrimental effects of OP exposure that actually existed when we do not have adequate power. Generally, although screening for the potential effects in the population displayed a high rate of false positives, it was still considered valuable because it greatly increased the likelihood of exploring the adverse health impact of neurotoxicants at an early age. The second strength was that all participants at 2 years of age had a homogenous socioeconomic profile, which helped us to reduce the potential impact from uncontrolled confounding factors.

The limitation that the variability of spot urine sample from each child must be considered, although some studies had confirmed that a single sample adequately predicted relative long-term average exposure (Meeker et al. 2005). Notably, the urinary DAP measures could imply both OP exposure and metabolites themselves in food and other environmental media (Lu et al. 2005). The study was also limited by the sensitivity and predictive validity of neurodevelopment assessment. The GDS had only modest predictive power for subsequent intelligence at school ages. In addition, the small sample size (*n* = 310) resulted in only a very small proportion of children (< 6%) with scores below the cutoff value of developmental delay, which led to less stable estimates of effect (wide confidence intervals). Finally, our findings should be interpreted with caution because DAP metabolites were not significantly associated with decreases in DQ scores. Similarly, Rauh et al. (2006) also found that although the actual difference in MDI scores for high versus low OP exposure group differed by only a few points (*p* > 0.05), the odds of highly exposed children having mental delays were 2.4 times greater than those in the low-exposure group (*p* < 0.05). Overall, larger sample size and more developmental assessment at older ages are needed to explore potential OP effects and possible dose–response relationships in the future.

To our knowledge, this is the first study to assess both prenatal and postnatal OP exposure in relation to infant neurodevelopment in a Chinese rural population. Despite the small sample size, the findings could arouse concern regarding infant health in a developing country. According to the GDS, 1–6% of the subjects in our study, as well as the investigated Shanghai children in Guodong et al.’s (2012) study, suffered from developmental delay at 2 years of age. Taking into account that less formal intervention services were provided to children who were at potential risk for early school failure in China, our study suggested that rural infants in a developing country deserve further protection from pesticides exposure, early identification of developmental deficits, and early developmental intervention.

## Conclusions

Both prenatal and postnatal exposure to OP pesticides may adversely affect the neurodevelopment of 2-year-old infants from the agricultural community. The present study adds to the accumulating evidence on associations of prenatal and postnatal OP exposure with infant neurodevelopment. These findings suggest that it is necessary to reduce both maternal exposure and children’s exposure to OP pesticides in the agricultural area.

## Supplemental Material

(196 KB) PDF
