# Supplementary material for: Adverse Associations of both Prenatal and Postnatal Exposure to Organophosphorous Pesticides with Infant Neurodevelopment in an Agricultural Area of Jiangsu Province, China
Source: Environ Health Perspect. 2016 May 6;124(10):1637–43. doi: 10.1289/EHP196 (PMC5047773; doi:10.1289/EHP196)
Supplement: (196 KB) PDF [file EHP196.s001.acco.pdf]

Environ Health Perspect
DOI: 10.1289/EHP196

Note to readers with disabilities: *EHP* strives to ensure that all journal content is accessible to all readers. However, some figures and Supplemental Material published in *EHP* articles may not conform to 508 standards due to the complexity of the information being presented. If you need assistance accessing journal content, please contact ehp508@niehs.nih.gov. Our staff will work with you to assess and meet your accessibility needs within 3 working days.

Supplemental Material

Adverse Associations of both Prenatal and Postnatal Exposure to Organophosphorous Pesticides with Infant Neurodevelopment in an Agricultural Area of Jiangsu Province, China

Ping Liu, Chunhua Wu, Xiuli Chang, Xiaojuan Qi, Minglan Zheng, and Zhijun Zhou

Table of Contents

Table S1. The distributions of maternal and child urinary DAP concentrations (nmol/L)

Table S2. DQ scores and proportions delayed according to the GDS (*n*=310)

Table S3. Association of maternal urinary DAP metabolites during pregnancy with fetal growth (*n*=310)

Table S4. Change in DQ scores in children tested at 2 years of age, for a 10-fold increase in maternal DAP concentrations during pregnancy (nmol/L)

Table S5. Change in DQ scores in children tested at 2 years of age, for a 10-fold increase in postnatal DAP concentrations (nmol/L)

Table S6. The comparison of the associations between DQ scores and total DAP concentrations measured in the prenatal vs. postnatal periods.

Table S1. The distributions of maternal and child urinary DAP concentrations (nmol/L)

Samples	Metabolites	LOD	Detection rate (%)	GM	GSD	Min.	Percentiles				Max.
							P25	P50	P75	P95	
Maternal urines before delivery	DMP	0.50	69.03	7.71	2.63	<LOD	<LOD	6.80	12.97	47.00	615.82
	DMTP	0.50	97.10	57.26	3.32	<LOD	26.26	62.28	157.64	276.01	675.15
	DMDTP	0.50	91.94	25.60	3.10	<LOD	12.18	27.25	55.08	155.83	409.96
	DEP	0.50	99.35	71.90	2.58	<LOD	40.52	75.73	136.19	291.28	1671.48
	DETP	0.25	88.71	18.90	4.68	<LOD	7.99	21.69	52.73	227.15	1719.79
	DEDTP	0.25	80.00	7.50	4.16	<LOD	3.42	6.73	17.70	84.14	211.79
	ΣDMs			120.66	2.26	<LOD	70.10	128.16	222.50	370.97	900.01
	ΣDEs			125.81	2.49	<LOD	75.58	134.88	229.21	491.23	1803.40
	ΣDAPs			277.79	1.98	<LOD	180.38	295.80	445.47	790.74	2144.77
Child urines at two years of age	DMP	0.50	76.10	17.07	4.19	<LOD	4.64	17.06	42.77	243.03	711.33
	DMTP	0.50	84.50	42.07	5.03	<LOD	14.53	49.86	128.09	488.20	2284.34
	DMDTP	0.50	88.70	44.39	4.52	<LOD	18.48	54.98	146.29	368.48	598.78
	DEP	0.50	90.60	25.75	3.29	<LOD	15.59	20.43	52.96	259.20	566.52
	DETP	0.25	91.30	45.36	4.87	<LOD	21.74	60.62	135.40	445.97	2796.77
	DEDTP	0.25	58.10	8.75	7.84	<LOD	<LOD	12.73	51.69	183.08	1864.08
	ΣDMs			163.51	2.98	<LOD	78.99	169.57	400.07	745.15	2528.11
	ΣDEs			126.02	3.08	<LOD	66.29	136.46	271.84	630.75	2800.02
	ΣDAPs			322.26	2.66	<LOD	169.83	350.41	687.80	1281.68	2967.94

LOD: limit of detection; GM: geometric mean; GSD: geometric standard deviation.

Table S2. DQ scores and proportions delayed according to the GDS (*n*=310)

Domains	Mean ± SD (range)	Normal[a] (%)	Developmental delay[b] (%)	Moderate delay[c] (%)	Severe delay[d] (%)
Motor area					
Male	99.11±8.43(54.17-130.43)	175(98.31)	3(1.69)	2(1.12)	1(0.56)
Female	99.52±9.37(54.17-130.43)	130(98.48)	2(1.52)	0(0.00)	2(1.52)
Total	99.28±8.83(54.17-130.43)	305(98.39)	5(1.61)	2(0.65)	3(0.97)
Adaptive area					
Male	96.11±7.98(62.50-126.09)	173(97.19)	5(2.81)	4(2.25)	1(0.56)
Female	96.26±8.54(54.17-113.04)	127(96.21)	5(3.79)	3(2.27)	2(1.52)
Total	96.17±8.21(54.17-126.09)	300(96.77)	10(3.23)	7(2.26)	3(0.97)
Language area					
Male	95.56±8.99(62.50-126.09)	169(94.94)	9(5.06)	7(3.93)	2(1.12)
Female	95.85±9.56(54.17-126.09)	123(93.18)	9(6.82)	6(4.55)	3(2.27)
Total	95.69±9.22(54.17-126.09)	292(94.19)	18(5.81)	13(4.19)	5(1.61)
Social area					
Male	96.14±8.14(62.50-130.43)	170(95.51)	8(4.49)	6(3.37)	2(1.12)
Female	98.10±8.43(54.17-121.74)	129(97.73)	3(2.27)	1(0.76)	2(1.52)
Total	96.97±8.31(54.17-130.43)	299(96.45)	11(3.55)	7(2.26)	4(1.29)
Average scores					
Male	96.73±7.50(65.63-128.26)	173(97.19)	5(2.81)	4(2.25)	1(0.56)
Female	97.43±8.18(54.17-118.48)	129(97.73)	3(2.27)	1(0.76)	2(1.52)
Total	97.03±7.80(54.17-128.26)	302(97.42)	8(2.58)	5(1.61)	3(0.97)

[a]Normal: DQ scores > 84

[b]Developmental delay: DQ scores ≤ 84

[c]Moderate delay: 70≤ DQ scores ≤ 84

[d]Severe delay: DQ scores < 70

Table S3. Association of maternal urinary DAP metabolites during pregnancy with fetal growth[a] (*n*=310)

Fetal growth	β (95% CI)	*p*	*p*-Value for interaction[b]	Male		Female	
				β (95% CI)	*p*	β (95% CI)	*p*
Birth weight (kg)							
DMs	-0.05(-0.19,0.09)	0.498	0.737	-0.07(-0.28,0.14)	0.534	-0.03(-0.22,0.16)	0.765
DEs	0.00(-0.13,0.14)	0.963	0.729	-0.01(-0.20,0.18)	0.894	0.06(-0.14,0.26)	0.567
DAPs	-0.07(-0.24,0.10)	0.410	0.661	-0.10(-0.35,0.16)	0.445	-0.02(-0.26,0.23)	0.898
Body length (cm)							
DMs	-0.19(-0.96,0.57)	0.620	0.171	0.51(-0.62,1.63)	0.376	-0.62(-1.68,0.45)	0.256
DEs	-0.42(-1.16,0.32)	0.269	0.854	-0.78(-1.80,0.23)	0.130	0.13(-1.03,1.28)	0.828
DAPs	-0.47(-1.43,0.49)	0.337	0.465	-0.29(-1.66,1.08)	0.674	-0.34(-1.76,1.08)	0.637
Head circumference (cm)							
DMs	-0.41(-0.86,0.03)	0.068	0.253	-0.65(-1.34,0.05)	0.067	-0.17(-0.75,0.41)	0.564
DEs	-0.36(-0.79,0.07)	0.102	0.406	-0.65(-1.29,-0.02)	0.043	-0.03(-0.66,0.60)	0.918
DAPs	-0.67(-1.22,-0.12)	0.017	0.307	-1.04(-1.88,-0.21)	0.015	-0.28(-1.05,0.50)	0.478
Neonatal PI							
DMs	-0.18(-1.38,1.01)	0.764	0.151	-1.26(-2.91,0.40)	0.137	0.49(-1.26,2.24)	0.580
DEs	0.60(-0.56,1.75)	0.310	0.824	0.93(-0.58,2.44)	0.226	0.26(-1.62,2.15)	0.784
DAPs	0.03(-1.47,1.52)	0.970	0.317	-0.41(-2.44,1.63)	0.694	0.15(-2.17,2.48)	0.897

[a]Adjusted for maternal age, gestational age, pregnancy weight gain, maternal BMI before pregnancy, parity, delivery mode, child's sex, passive smoking, maternal work status, paternal work status, family annual income, maternal education, cord blood lead values, sampling season and inhabitation.

[b] Interactions between DAP concentrations and child's sex.

Table S4. Change in DQ scores in children tested at 2 years of age, for a 10-fold increase in maternal DAP concentrations during pregnancy (nmol/L)[a]

Domains	β (95% CI)	*p*	*p*-Value for interaction[b]	Male		Female	
				β (95% CI)	*p*	β (95% CI)	*p*
Motor area							
DMs	0.98(-1.89,3.85)	0.501	0.375	-0.01(-4.11,4.08)	0.994	2.15(-2.15,6.45)	0.324
DEs	0.53(-2.17,3.23)	0.700	0.781	1.12(-2.46,4.70)	0.537	0.22(-4.31,4.76)	0.922
DAPs	1.08(-2.45,4.61)	0.547	0.912	1.40(-3.48,6.29)	0.572	1.61(-4.05,7.23)	0.575
Adaptive area							
DMs	-0.22(-2.87,2.42)	0.867	0.521	1.38(-2.41,5.17)	0.472	-1.55(-5.44,2.34)	0.431
DEs	-0.25(-2.73,2.23)	0.844	0.689	0.30(-3.03,3.62)	0.861	-1.50(-5.59,2.58)	0.467
DAPs	-0.99(-4.23,2.26)	0.551	0.475	0.72(-3.82,5.25)	0.755	-3.14(-8.22,1.93)	0.223
Language area							
DMs	-0.23(-3.19,2.74)	0.882	0.334	1.71(-2.65,6.07)	0.440	-1.98(-6.26,2.31)	0.362
DEs	0.62(-2.17,3.40)	0.663	0.590	0.98(-2.85,4.80)	0.614	-0.75(-5.25,3.76)	0.744
DAPs	0.17(-3.48,3.82)	0.927	0.379	1.60(-3.61,6.82)	0.544	-2.25(-7.87,3.37)	0.430
Social area							
DMs	-0.44(-3.10,2.23)	0.748	0.880	0.30(-3.59,4.19)	0.879	-0.91(-4.82,3.01)	0.647
DEs	0.97(-1.53,3.47)	0.444	0.898	1.63(-1.77,5.02)	0.345	-0.22(-4.33,3.88)	0.914
DAPs	0.09(-3.18,3.37)	0.956	0.707	1.59(-3.04,6.23)	0.499	-1.52(-6.64,3.61)	0.559
Average scores							
DMs	0.03(-2.49,2.54)	0.985	0.810	0.85(-2.77,4.46)	0.645	-0.57(-4.33,3.19)	0.764
DEs	0.47(-1.89,2.83)	0.697	0.706	1.01(-2.15,4.17)	0.531	-0.56(-4.51,3.38)	0.778
DAPs	0.09(-3.00,3.18)	0.955	0.606	1.33(-2.98,5.64)	0.544	-1.33(-6.25,3.60)	0.595

[a]Adjusted for maternal age, gestational age, pregnancy weight gain, maternal BMI before pregnancy, parity, delivery mode, child's sex, passive smoking, maternal work status during pregnancy, paternal work status, family annual income, maternal education, cord blood lead values, sampling season and inhabitation.

[b]Interactions between DAP concentrations and child's sex.

Table S5. Change in DQ scores in children tested at 2 years of age, for a 10-fold increase in postnatal DAP concentrations (nmol/L)[a]

Domains	β (95% CI)	*p*	*p*-Value for interaction[b]	Male		Female	
				β (95% CI)	*p*	β (95% CI)	*p*
Motor area							
DMs	0.40(-1.70,2.50)	0.713	0.881	0.77(-1.96,3.49)	0.578	0.93(-2.66,4.51)	0.609
DEs	0.27(-1.82,2.36)	0.797	0.455	-0.11(-2.81,2.60)	0.938	1.66(-1.86,5.19)	0.352
DAPs	0.44(-1.91,2.78)	0.715	0.691	0.33(-2.68,3.34)	0.831	1.70(-2.33,5.72)	0.405
Adaptive area							
DMs	0.31(-1.52,2.14)	0.740	0.653	1.24(-1.09,3.56)	0.295	0.90(-2.14,3,95)	0.558
DEs	0.01(-1.82,1.83)	0.996	0.842	0.24(-2.07,2.55)	0.838	0.45(-2.56,3.46)	0.768
DAPs	0.19(-1.86,2.23)	0.859	0.933	0.86(-1.71,3.43)	0.511	0.72(-2.71,4.15)	0.680
Language area							
DMs	-0.15(-2.23,1.93)	0.886	0.856	0.22(-2.53,2.97)	0.874	0.66(-2.75,4.08)	0.701
DEs	-0.37(-2.44,1.70)	0.722	0.977	-0.21(-2.94,2.53)	0.882	0.04(-3.33,3.41)	0.981
DAPs	-0.24(-2.57,2.09)	0.840	0.973	0.03(-3.01,3.07)	0.987	0.54(-3.30,4.38)	0.780
Social area							
DMs	-0.76(-2.61,1.10)	0.422	0.829	-0.18(-2.58,2.22)	0.881	0.21(-2.82,3.24)	0.892
DEs	-1.15(-2.99,0.70)	0.222	0.871	-1.18(-3.55,1.20)	0.330	-0.36(-3.34,2.63)	0.814
DAPs	-1.08(-3.15,0.99)	0.307	0.959	-0.76(-3.40,1.89)	0.574	-0.09(-3.50,3.32)	0.958
Average scores							
DMs	-0.05(-1.81,1.71)	0.955	0.785	0.51(-1.74,2.76)	0.655	0.68(-2.25,3.60)	0.648
DEs	-0.31(-2.06,1.44)	0.726	0.757	-0.31(-2.55,1.92)	0.783	0.45(-2.44,3.33)	0.758
DAPs	-0.17(-2.14,1.79)	0.862	0.920	0.11(-2.37,2.60)	0.928	0.72(-2.57,4.01)	0.667

[a]Adjusted for child's sex, feeding pattern, inhabitation, child hand-to-mouth contacts, whether families lived nearby plantations or green parks, whether chipped paint falling from the wall in homes, passive smoking, whether family used indoor insecticides within one year, whether family used mosquitocides within one year, at least one farmer in household, family annual income, maternal work status, paternal work status, maternal education and sampling season.

[b] Interactions between DAP concentrations and child's sex.

Table S6. The comparison of the associations between DQ scores and total DAP concentrations measured in the prenatal vs. postnatal periods.

Domains	All			Male			Female		
	prenatal β (95% CI)	postnatal β (95% CI)	p^a	prenatal β (95% CI)	postnatal β (95% CI)	p^a	prenatal β (95% CI)	postnatal β (95% CI)	p^a
Motor area	0.03(-1.29,1.35)	0.03(-0.83,0.90)	0.998	0.05(-2.06,2.16)	0.01(-1.27,1.28)	0.973	-0.06(-2.51,2.39)	0.32(-1.45,2.08)	0.810
Adaptive area	-0.14(-1.57,1.30)	0.04(-0.86,0.93)	0.838	-0.13(-2.50,2.25)	0.19(-1.13,1.51)	0.813	-0.67(-3.30,1.95)	0.20(-1.60,1.99)	0.596
Language area	-0.09(-1.57,1.39)	-0.02(-0.95,0.91)	0.935	-0.03(-2.31,2.25)	-0.02(-1.33,1.28)	0.999	-0.59(-3.55,2.36)	0.23(-1.81,2.26)	0.658
Social area	-0.13(-1.62,1.36)	-0.17(-1.10,0.76)	0.965	-0.17(-2.63,2.29)	-0.20(-1.58,1.18)	0.981	-0.38(-3.00,2.23)	0.04(-1.72,1.80)	0.794
Average scores	-0.09(-1.43,1.25)	-0.02(-0.86,0.82)	0.927	-0.07(-2.22,2.08)	-0.00(-1.22,1.21)	0.956	-0.43(-2.92,2.05)	0.24(-1.44,1.92)	0.662

$^a p$: For testing whether the effect coefficients of urinary DAPs measured in the prenatal vs. postnatal periods were significantly different.
